# “A time of fear”: local, national, and international responses to a large Ebola outbreak in Uganda

**DOI:** 10.1186/1744-8603-8-15

**Published:** 2012-06-13

**Authors:** John Kinsman

**Affiliations:** 1 Umeå Centre for Global Health Research, Epidemiology and Global Health Unit, Department of Public Health and Clinical Medicine, Umeå University, 901 85, Umeå, Sweden

**Keywords:** Ebola, Outbreak, Uganda, Fear, Stigma, Altruism

## Abstract

**Background:**

This paper documents and analyses some of the responses to the largest Ebola outbreak on record, which took place in Uganda between September 2000 and February 2001. Four hundred and twenty five people developed clinical symptoms in three geographically distinct parts of the country (Gulu, Masindi, and Mbarara), of whom 224 (53%) died. Given the focus of previous social scientific Ebola research on experiences in communities that have been directly affected, this article expands the lens to include responses to the outbreak in local, national, and international contexts over the course of the outbreak.

**Methods:**

Responses to the outbreak were gauged through the articles, editorials, cartoons, and letters that were published in the country’s two main English language daily national newspapers: the New Vision and the Monitor (now the Daily Monitor). All the relevant pieces from these two sources over the course of the epidemic were cut out, entered onto a computer, and the originals filed. The three *a priori* codes, based on the local, national, and international levels, were expanded into six, to include issues that emerged inductively during analysis. The data within each code were subsequently worked into coherent, chronological narratives.

**Results:**

A total of 639 cuttings were included in the analysis. Strong and varied responses to the outbreak were identified from across the globe. These included, among others: confusion, anger, and serious stigma in affected communities; medical staff working themselves to exhaustion, with some quitting their posts; patients fleeing from hospitals; calls on spiritual forces for protection against infection; a well-coordinated national control strategy; and the imposition of some international travel restrictions. Responses varied both quantitatively and qualitatively according to the level (i.e. local, national, or international) at which they were manifested.

**Conclusions:**

The Ugandan experience of 2000/2001 demonstrates that responses to an Ebola outbreak can be very dramatic, but perhaps disproportionate to the actual danger presented. An important objective for any future outbreak control strategy must be to prevent excessive fear, which, it is expected, would reduce stigma and other negative outcomes. To this end, the value of openness in the provision of public information, and critically, of being *seen* to be open, cannot be overstated.

## Background

The gruesome death that frequently accompanies Ebola Haemorrhagic Fever has ensured that the disease is etched deeply into the public imagination. Initially, an Ebola patient is likely to complain of non-specific symptoms such as high fever, weakness, diarrhoea, nausea, headache and vomiting. However, their condition can deteriorate quickly and dramatically, to include rashes, impaired kidney and liver function, and in some cases, both internal and external bleeding [1]. Those who die do so usually within two weeks of disease onset, often having exhibited the classical “deep-set eyes, ghost-like expressionless face, and extreme lethargy” [2].

Although it is rare, Ebola has a high case fatality rate. Just 2,306 cases have been reported since the disease was first recognised in 1976 in Zaire (now the Democratic Republic of Congo, or DRC), but 1,527 (66%) of these individuals died [3]. There remains no specific therapy, and neither is there a vaccine [4], although recent experimental vaccine studies have shown some promise [5].

Ebola is a zoonotic disease: bats are currently suspected as the most likely reservoir species for the virus [4]. Outbreaks occur when an individual comes into contact with an infected animal, with subsequent transmission between humans taking place through direct contact with the blood and/or secretions of an infected person. Whole families can therefore be infected when they are caring for a sick relative, or when preparing a body for burial. Serious amplification of an epidemic can also occur in hospitals and clinics if protective clothing is not worn by health care workers, or if needles and syringes are not sterile [6].

All of the known Ebola outbreaks have originated in tropical African countries, including Sudan, Zaire/DRC, the Republic of Congo, Gabon, Côte d’Ivoire, and Uganda. Because of the speed with which Ebola kills, outbreaks tend to be self-limiting. Indeed, there is evidence that sporadic and unrecognised, or misdiagnosed outbreaks with low levels of secondary transmission, may occur relatively frequently [7]. Nonetheless, containment measures including early hospitalisation and isolation, disposal of all materials that come into contact with victims, barrier nursing methods, immediate burial of the dead, provision of health education messages, and active surveillance can significantly reduce the number of infections and deaths [8,9].

The largest Ebola outbreak on record first emerged in the environs of Lacor Hospital in the northern Ugandan town of Gulu, in September 2000. Unidentified for some time, the hospital’s Medical Superintendent reported an “unusual severe febrile illness” to the Ministry of Health on October 8. An isolation ward was established at the hospital two days later, and the outbreak was subsequently confirmed as Ebola by a laboratory at the South African National Institute of Virology on October 15 [10].

**Figure 1:**
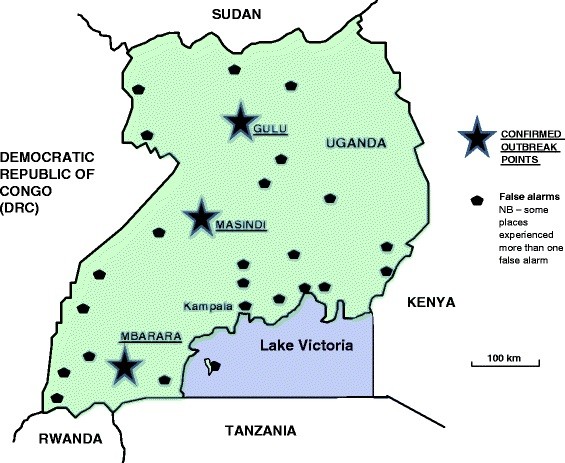
Map of Uganda showing confirmed Ebola outbreak points, and locations where false alarms were sounded.

A total of 425 clinical cases were reported during this outbreak, of whom 224 (53%) died. Three districts were affected, two in the centre-north of the country, and one far away in the south west (see Figure 1). Significantly more females than males were infected (female:male ratio = 63:37), largely because women generally take on greater responsibility than men for caring for the sick and preparing bodies for burial [11,12]. In addition, 31 health workers contracted the virus (7% of the total number infected), of whom 17 died. Uganda was finally declared Ebola-free on February 27, 2001 [13]. 

As with all previous Ebola outbreaks, most of the literature on this case focused on clinical, epidemiological, and control issues. Indeed, to date, just a few social scientific Ebola studies have been conducted [12,14,15], and these have tended to examine how people in the affected communities perceived and responded to the outbreaks. Relatively little attention has been given to events from national, regional, or international perspectives. The objective of this paper, therefore, is to explore, on a more holistic basis, some of the responses to the large 2000/2001 Ebola outbreak in Uganda. In so doing, it reflects on the ways in which three important human experiences – fear, altruism, and stigma – were felt and enacted by people throughout Uganda, as well as in countries many thousands of kilometres away.

### Social responses to Ebola

The outbreak of a deadly infectious disease sets in motion two parallel processes. One of these is epidemiological, in which investigations are undertaken by the authorities to establish what is the responsible aetiological agent, who is at risk of infection, through which activities, and what can be done to reduce that risk [16]. With this information, an epidemiological picture of the outbreak is produced, and appropriate containment and control strategies can then be set in place.

The other process is social. In previous outbreaks, Ebola had a profound psychological effect on its victims, the health workers taking care of them, as well as the affected communities. “Alarm and near panic” were reported among health workers at Maridi hospital in Sudan in 1976 during the second ever recorded outbreak, an understandable condition given that 61 of the hospital’s 154 nursing staff had fallen ill, of whom 33 then died [2]. During the 1995 epidemic in Kikwit, Zaire, health workers constituted 25% of the 315 Ebola cases [8], and fear of infection led many of them to quit their posts. Furthermore, those who bravely stayed at work subsequently reported feeling stigmatised, because many people feared that they might act as carriers of the virus into the wider community. In some cases, neighbours threw stones at them, while others were chased from their houses [17].

One way or another, these responses were all predicated on one core factor: fear. Defined as “a feeling of apprehension or alarm in response to an external source of danger” [18], and often manifested in these outbreaks as panic, this fear also contributed to two broad categories of behaviour that were directed towards the people and groups who were affected and infected by Ebola. These response categories were altruism and stigma.

Different scientific disciplines have taken different approaches to defining altruism, but perhaps the most meaningful definition for this study is as an act that “is or appears to be motivated mainly out of a consideration of another’s needs rather than one’s own” (page 30) [19]. Health workers were the most obviously altruistic people in these outbreaks, but the fact that the altruistic act can appear to be motivated *mainly* by selfless considerations also allows some degree of self-interest to be included in the analysis. In other words, an altruistic act may be ‘selflessly’ motivated by a humanitarian imperative, or, otherwise put, by a strongly felt need to act against a fearsome onslaught of some sort. But a secondary set of more self-interested motivations may also be identified, based on a (fear-driven) desire to avoid an unwelcome material outcome, such as losing a job; or on a wish to earn some sort of financial or social gain.

Stigma is, of course, an entirely different sort of response. In his seminal work on the subject, Goffman defined a stigmatised individual as one with an “attribute that is deeply discrediting”, and who is thereby “reduced in our minds from a whole and usual person to a tainted, discounted one… [to the point where] we believe the person with the stigma is not quite human. On this assumption we exercise varieties of discrimination, through which we effectively, if often unthinkingly, reduce his life chances” (pages 3–5) [20]. This reduction in life chances is effected through the labelling of an individual or a group as different or deviant, thus producing a state, ultimately founded on fear, of ‘us’ and ‘them’. If ‘they’ have less power than ‘us’, a loss in status or some other form of discrimination may then follow [21].

## Methods

Responses to the outbreak were gauged through the articles, editorials, cartoons, and letters that were published in the country’s two main English language daily national newspapers: the New Vision and the Monitor (now the Daily Monitor). The New Vision is generally supportive of the Ugandan government, while the Monitor provides a more independent perspective. Both sources were treated equally in the analysis, with no more weight being given to articles from either the New Vision or the Monitor.

All the relevant pieces from these two sources – between October 13 2000, when the first reports of a “strange disease” appeared, and February 27 2001, when the epidemic was officially declared over – were cut out on a day-by-day basis as the outbreak unfolded, entered onto computer, and the originals filed. Following the principles of thematic analysis, the data were then moved as appropriate into three *a priori* codes. These were based on the multi-level framework used in the study: (i) responses in the affected communities (i.e. including material from all three confirmed outbreak sites), (ii) responses throughout the country, and (iii) international level responses. Reviewing the data within this format, it became clear that three additional codes would be required for the data coded under ‘responses throughout the country’: these included responses of the medical fraternity, the government’s response, and controversy over the source of the outbreak. Folders for these three codes were therefore created, and the relevant material was moved in. The most illustrative anecdotes and quotes from each of the six thematic codes were then extracted, and these were worked into coherent, chronological narratives.

## Results

**Figure 2:**
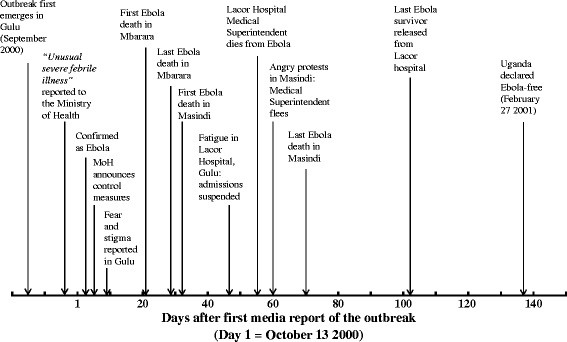
Timeline showing some of the major events from the Ugandan Ebola outbreak.

A total of 639 cuttings were taken, 371 (58%) of which came from the New Vision and 268 (42%) from the Monitor. This represented a mean of 2.7 and 1.9 cuttings per day respectively. The cuttings included 539 articles, 38 letters to the Editor, 34 photographs (independent of an accompanying article), 15 editorials and commentaries and 13 cartoons. There were two peaks in coverage by the two newspapers. The first came in Week 2, by when the potential seriousness of the outbreak had been fully recognised – 77 cuttings were taken. The second, larger peak was in Week 9, following the death from Ebola of the man who had first alerted the Ministry of Health about the outbreak, Dr Matthew Lukwiya, Medical Superintendent of Lacor Hospital – 102 cuttings were taken. Otherwise, there was a consistent and gradual decline in coverage from Week 2 onwards. Some of the major events of the outbreak are presented in Figure 2, in order to give a broad overview of the story as it unfolded. More of the day-to-day detail is given in Additional file 1: Annex 1, which lists the headlines of each of the 88 newspaper articles quoted in the text below.

Because it was not always possible to establish from the reports exactly when a given incident had taken place, the day numbers below refer to the day on which the particular stories were published. The first report of the disease was published in the New Vision on October 13, and this is taken as the reference point, or Day 1.

### Responses in the affected communities

#### Gulu: Epicentre of the outbreak

At the time of the outbreak, Gulu district had been at the centre of a brutal rebel insurgency by the Lord’s Resistance Army (LRA) for 13 years, ongoing since 1987. The community’s response to Ebola there should be seen in this context. Just three days before the Ebola outbreak was officially announced, for example, 11 people were killed and over 50 injured in three separate attacks on Gulu town during Independence Day celebrations. During the outbreak itself, health workers and scouts travelling to affected villages were obliged to travel in Uganda People’s Defence Force (UPDF) armoured personnel carriers in order to avoid the rebels. One report from Day 7 described the visit of a medical team under heavy military escort to five patients trapped at their homes. UPDF protection was not, however, always a guarantee of safety. One Ebola surveillance scout was killed during an attack on Day 31.

Gulu bore the brunt of Uganda’s Ebola epidemic, with 393 cases (92% of the total) and 203 deaths [13]. The Medical Superintendent at Gulu hospital described the situation as “very wild” on Day 2, adding that two suspected cases had fled the hospital. Four days later, mourners at the funeral of an Ebola victim stripped themselves naked and left their clothes for fear of carrying the virus home with them, to which the Minister of Health responded, “sometimes the response is extreme, but it shows that people are taking the message.” Unfortunately, a number of people took the wrong message, drinking and bathing in ‘Jik’ bleach (which was heavily promoted throughout the epidemic as an effective disinfectant against the virus) “in a desperate attempt to rid themselves of Ebola”.

Relations between people within the community rapidly deteriorated as the infection spread. A reporter in Rwot-Obillo, one of the first affected villages, described “people eyeing each other suspiciously, suspecting everybody else to be a carrier of the killer virus”, while inhabitants of Gulu town were said to be “dispirited, suspicious and depressed”. People refused to shake hands, they avoided gatherings, markets and day schools were closed, and all visits to boarding schools were banned. One head teacher explained that “the teachers monitor the children very closely. We tell them to avoid touching each other. We also instruct them to play games where there is no body contact”.

On Day 19, Gulu district “erupted in wild shouting, jumping and running”, as a traditional Acholi ritual was performed, intended to “exorcise” their land of the virus. The procedure, known as *ryemo gemo*, is practiced every December 31 as a means of cleansing the area of disease before the New Year begins [12]; but this additional *ryemo gemo* ceremony – in which the participants “carried spears and ran around beating saucepans, basins, calabashes, and jerry cans” – was conducted in late October in the hope that their actions would chase Ebola across the Nile and away.

Meanwhile, some traditional healers had told people to tie banana fibres around their wrists as a means of avoiding Ebola. However, most healers had suspended curative work in the early stages of the outbreak, since a number of their colleagues had initially thought that they would be able to treat Ebola, but they had contracted the virus during their attempts and succumbed themselves. The Roman Catholic clergy responded by visiting many of the affected villages to tell people “to ignore such witchcraft”. As one priest said wearily, “because it is a time of fear, people resort to superstitious practices.”

Stigmatisation rapidly became a major issue, just as it had done in the Kikwit outbreak of 1995. On Day 5, Dr Lukwiya of Lacor Hospital said, “Once the names of the Ebola patients and the dead are made public, society stigmatises the relatives”. Considerable difficulties were also faced after the epidemic when trying to resettle the 190 survivors back into their communities, even though they had fully recovered. Returning home, many found that fearful neighbours had burned their blankets, clothes, beds, and in some cases even their huts. Others discovered that their spouses had fled, and they were completely shunned by their neighbours. People refused to buy from Ebola survivors, so that those who had previously worked in the markets found themselves without any means of support. However, a concerted education programme was launched which eventually ensured that survivors could once again join the community. As one woman said, “they burned all my things. I am left with nothing except the clothes I am wearing now. Even family members at first feared to mix with me, but I told them the doctors said it is OK. Now relatives have started coming back.”

Because traditional burial practices had been a major factor in initial amplification of the epidemic, the authorities were obliged to take over responsibility for burying the dead. Trained teams buried 138 of the victims in one cemetery just outside Gulu town, but the community as a whole was not able to properly mourn its dead until the day the epidemic was officially declared over, February 27 2001. Hundreds of relatives gathered at the cemetery on that day, many of them “sitting on the graves, crying and rolling on the ground, dust and pieces of dry grass stuck to their hair and clothes, even as the two bishops and a Muslim leader began prayers.”

#### Mbarara: A small, controlled outbreak within the army

The Mbarara outbreak struck only five individuals, of whom four died. Starting on Day 21, it took both the authorities and the public by surprise, since few travellers move directly between there and Gulu, over 500 km to the north, which was at that time the only other place suffering an outbreak. The first case was a UPDF Private at the barracks in Mbarara town, and the other four cases were either directly or indirectly connected to the army, which had the means to impose strict and immediate control measures. As a result, the outbreak remained within this small closed circle of colleagues, so community reaction never reached the same pitch as it did either in Gulu or Masindi (see below for discussion of events in Masindi). The outbreak was short-lived, with the fourth and final death taking place on Day 29.

There was, however, some anger over the choice of burial site for the victims. A Local Councillor said during a phone-in radio show that was reported in the Monitor: “Enough is enough. We reject being the Municipality’s garbage dump and we will attack the team if they bring another Ebola body here”. Listeners who subsequently phoned in expressed support for this position, and some residents of Mbarara Municipality also threatened to attack the University teaching hospital if another victim was buried in their area. The newspapers never reported how this issue was resolved. Other reported reaction in Mbarara town was limited to an increase in the price of Jik, from 1,500 to 2,000 Uganda shillings (US$0.85 to US$1.15) per bottle, people avoiding shaking hands and hugging, and poor business at hotels and kiosks. “I still have almost all the food and tea we have prepared for breakfast and lunch”, said one food vendor on Day 23. “We don’t think we shall open tomorrow”.

#### Masindi: An angry community response

Just as events were being brought under control in Mbarara, the epidemic appeared 130 km south of Gulu town, in Kigumba village, Masindi district. On Day 32, the death was reported of a woman who had initially been admitted with abdominal swelling at Lacor Hospital in Gulu, and who had “escaped” from there when she learned that the hospital was also treating Ebola patients. Unaccountably infected while at Lacor, she carried the virus home and then infected three of the close relatives who cared for her when she eventually fell ill. The four of them all died. As per local custom, their bodies were kept for three days before burial, and were ritually and communally washed. A senior member of the National Ebola Task Force bemoaned the fact that “they refused to go to hospital, and they did not allow anyone to go to their homes. Kigumba is a very complex area,” he added. “It has many nationalities, and it is very deprived, without facilities and infrastructure.”

A burial team at nearby Kiryandongo hospital was rapidly trained by experts brought down from Gulu, and ten graves were prepared. Because it lacked a reliable power supply, running water and communication facilities, Kiryandongo was established only as a ‘holding point’ for suspected cases. Confirmed cases were to be transferred to the much better equipped Masindi hospital, 50 km away in the district capital. Nonetheless, the preparations at Kiryandongo prompted 33 “panic-stricken” patients, admitted with other conditions, to flee back into the community. Meanwhile, an Ebola isolation ward with eight beds was established in Masindi, which required discharging all the patients from a TB ward, including several prisoners who were to continue receiving treatment in their cells.

The death toll gradually grew, and by Day 55, 12 people had died and a further 12 were still admitted. Serious problems in the community first arose with the people who lived near to the official Ebola burial site, three kilometres outside of Masindi town. “Hundreds of angry villagers” took to the streets on Day 56, and stormed the district headquarters in protest, forcing officials to seek an alternative site. Meanwhile, the Ebola burial team abandoned work due to extreme pressure from the community, leaving four bodies unburied in the hospital, which in turn prompted 100 patients in other wards to flee. Residents in some areas of town began refusing medical workers at Masindi hospital access to their own homes, some of which were attacked. This prompted a press release from the Bunyoro-Kitara kingdom – which traditionally leads the people of Masindi – calling for tolerance. “Relatives have turned against their brothers and sisters who work at Masindi and Kiryandongo hospitals,” it stated. “A deep division has been created in the population”.

This statement, and the high level meeting of traditional leaders that followed it, did not, however, bring an end to the vitriol. The Chairman of the District Task Force received death threats from people who blamed him for the death from Ebola of the hospital’s ambulance driver, who had been infected while transporting patients from Kiryandongo to Masindi. On Day 60, the Medical Superintendent of Masindi Hospital decided that he too was at risk, and he fled with his family to Kampala, Uganda’s capital city. That same evening, two members of a relief burial team that had just arrived in Masindi from Gulu were unceremoniously thrown out of their hotel and threatened with death.

The Minister of State for Local Government then stepped in, pointing out that the Penal Code would be invoked to deal with those who took the law into their own hands. “All those harassing our dedicated health workers in the fight against Ebola should be condemned with the contempt they deserve”, he stated. The government also sought to tackle the problem at source, by strengthening the isolation centre at Kiryandongo so that it could handle cases that emerged there, while Masindi hospital would take on cases from elsewhere in the district. As it happened, the outbreak in Masindi was almost over by this stage. The last of the 17 deaths reported in the district occurred just over a week later, on Day 70.

### Responses throughout the country: Panic amid fears of divine retribution

There were numerous stories from throughout the country about reaction to the threat of Ebola. Forty-five false alarms were described in the newspapers (see Figure 1), arising out of a wide variety of conditions – dysentery, malaria, cholera, food poisoning, hypertension (which reportedly caused a nose bleed), gastro-enteritis, septic abortion, a gun shot wound, menstrual periods, excessive alcohol consumption, gingivitis, and haemorrhoids. The word “panic” accompanied 19 of these reports. In one case, the suspicious death of a man in a Kampala hospital sparked off a “stampede” in which workers and patients fled, while others were wheeled out of the ward or carried from their beds. Several people were reportedly injured during another chaotic scene in Jinja, when nurses “abandoned their desks and threw away their pens and writing pads”, while patients “scampered away” from the suspect individual. Meanwhile, a man in Iganga – 400 km away from the nearest confirmed case – barricaded himself and all his family inside their house with sufficient food and water to last until the outbreak ended, while bank clerks in Mbale started wearing latex gloves whenever they handled money.

Even petty criminal activity was affected, with pickpockets at a Kampala bus park shunning travellers who had just arrived either from Masindi or Gulu. Instead of approaching just anyone who looked like easy prey, the pickpockets sought first to establish where their target had come from. “They stand near the buses to make sure that the person they are going to steal from is not from Gulu or Masindi,” reported an anonymous source to the New Vision. Any pickpocket who accidentally came into contact with a passenger from an affected area was immediately “excommunicated” by his colleagues. One boy, for example, who had worked his hand into an old man’s bag when the latter informed him he was from Gulu, was “suspended from his group”.

Religion was for many people an important source of support, explanation and, they hoped, protection. On Day 29, over 300 Christian churches from throughout the country converged on a stadium in Kampala to pray for the end of the Ebola outbreak. Several priests took the opportunity to emphasise the need for people to follow the scriptures. As one of them said, “whenever there is famine, an epidemic or endless wars, the Bible says it is coming from God on a particular people or nation for sins committed”. This theme of divine retribution was echoed by the leader of the Muslim Tabliq sect on Day 61, who stated that “whenever man sins, such punishments like the deadly Ebola and AIDS attack mankind”.

Attempts at protection from the virus were also made in remote Kotido district in the east of the country, when, on Day 58, Karimojong warriors instigated an anti-Ebola ritual. According to the head master of a local school, this was “meant to cast out and cleanse the area of all bad omens, especially Ebola”. Several goats were killed and their intestines laid out on the ground in order to assess and interpret what lay in the future. The intestines of the first five goats apparently all read “negative”, which implied “imminent doom” for the Karimojong people. Consequently, 2000 participants in the ritual were then obliged to step on goat dung and smear their foreheads with it as “inoculation” against the virus.

### Responses of the medical fraternity: Courage, exhaustion, and the (occasional) abandonment of posts

On Day 9, a senior WHO official described the medical facilities in Uganda as “outstanding compared to the classic Ebola situation” – by which he referred to poorly equipped health centres in such places as rural Sudan or DRC – but the people working in these facilities were quickly stretched to breaking point. The intense and sustained concentration required to care safely for Ebola patients exhausted the health workers, and mistakes were made. Fourteen of the 22 health workers infected in Gulu and Lacor Hospitals acquired their infection after isolation wards and other containment measures had been established [10].

Lacor Hospital was probably the best equipped and best manned of all the centres involved, but it was obliged to take the drastic step of temporarily suspending all admissions on Day 47, on account of severe physical and mental fatigue among staff. Ten health workers had died there by that stage, six in the previous 10 days. A reporter described the atmosphere as “grim, stressful and dispiriting”, and threats were circulating of an imminent strike. The most devastating blow to morale took place on Day 55, when the Medical Superintendent, Dr Lukwiya himself, died from Ebola, which had the effect of “paralysing operations in the hospital”.

Meanwhile at Masindi hospital, a cleaner in the isolation unit and an ambulance driver had fallen ill on Day 54, prompting an outcry among their colleagues who were reportedly “frightened and demoralised.” On Day 58, Masindi hospital Medical Superintendent complained that some doctors and nurses had suddenly started demanding for sick or annual leave, while others were simply not turning up for work. Nurses were complaining of exhaustion, medics were few and overworked, and the burial team had abandoned duty after facing serious stigmatisation from the community.

Finally, there was some good news on Day 63, when it was announced that 30 clinical officers and nurses from other parts of the country had volunteered to work with Ebola patients in Masindi and Gulu to relieve the medical teams there. These volunteers were not, however, entirely representative of their colleagues nationwide. A series of interviews had been published on Day 59 quoting nurses from an unaffected part of eastern Uganda, a number of whom said they would refuse to treat any Ebola patient. As one stated, “If Ebola breaks out here, I will not risk my life attending to patients, not after all those other nurses and a hospital superintendent died. I will stay away from the hospital. Let me lose my job rather than losing my life.”

The very particular risks faced by health workers provoked a powerful wave of demands for compensation and risk allowances. On Day 55 – the day of Dr Lukwiya’s death – the Ministry of Health announced that it was initiating the compensation process for the families of all health workers who had died from Ebola. This was not enough for some MPs, however, who called for special payments to uninfected health workers. As one said, “Praising them when they are dead is not enough.” Consequently, the Director General of Health Services announced on Day 64 that staff working with Ebola would receive between 15,000 and 25,000 Uganda shillings (US$8.60 to US$14.30) extra allowance per day, depending on their job. The President himself also directed that the families of health workers who died from Ebola should be paid compensation equivalent to five months’ salary.

### The government’s response: Coordination, surveillance, and provision of information

Already the day before the Ministry of Health confirmed the outbreak to be Ebola, the Director General of Health Services issued a statement calling for special hygienic precautions when handling patients suspected to be suffering from this “strange” disease, as well as during the funerals of victims. On Day 4, the Minister of Health announced plans during a national radio and TV broadcast to recruit health scouts throughout Gulu district, to equip hospitals with medication and protective gear, and to establish national and district task forces that would meet daily. He added that people with symptoms should report at once for treatment, and that the dead should be buried immediately, but he also stressed that there was no need for panic. “I assure the people in Uganda and our international partners that there should be no cause for alarm, as the steps that government has taken are adequate to contain this outbreak of Ebola”.

The Ministry of Health co-ordinated the entire national control operation. This included establishing a highly sensitive surveillance system, whereby 150 volunteers in Gulu alone followed up 5,600 contacts for 21 days each (the maximum incubation period of the virus). Other activities involved updating hospital control measures, establishing safe-burial teams, and community education. The Ministry also requested WHO to co-ordinate the international response [10]. Once the epidemic was brought under control, providing support for the 600 Ebola orphans and 201 survivors became a priority. The Ministry distributed 70,000 Uganda Shillings (US$40) to each survivor in order to replace personal effects destroyed by neighbours while they were ill. It also co-ordinated the efforts of various Non Governmental Organisations to assist orphans, as well as distributing supplies donated by private organisations.

Providing assurances and accurate information to an increasingly concerned public was one of the most important components of the official response. As an opinion column in the Monitor pointed out, “Mass hysteria is best managed with calmness and scientific facts”. Frequent announcements from the Ministry of Health in all Uganda’s languages were therefore made over the radio, concerned with prevention and care, the development of the epidemic, and control measures in all the affected areas. However, there were cases of senior officials expressing perhaps unjustified optimism, which demonstrated the difficulty of finding a balance between being entirely honest and trying to keep people from becoming unduly anxious. For example, one individual stated on Day 8 that “We will get on top of this disease, and in a week or two, new cases will be history.” When this clearly did not happen, some people began to suspect that the government was not telling the truth about the numbers of infected people, and perhaps more crucially, whether or not the virus had spread to Kampala. On Day 58, a member of the National Task Force felt obliged to address this issue, stating, “I am on the ground in the whole of Kampala and I am not aware of any Ebola case. If we hide Ebola, we will kill ourselves. We are very open.”

Unfortunately, this openness did not in all cases translate to officials wanting to take active responsibility. A row erupted on Day 68 between Kampala City Council (KCC) and the Police over who should remove the corpse of an old man suspected to have died from Ebola. The man had reportedly been bleeding from the nose, eyes and mouth before he died at the entrance to one of Kampala’s taxi parks, and neither group wanted to take responsibility for the case. The Regional Police Commander said “KCC has a dumper and is equipped to handle Ebola cases”, while the KCC task force in turn said that since there was no Ebola in Kampala, it was the police’s responsibility to take the body away. The corpse lay in the road for 36 hours before a team hired by KCC finally picked it up.

In accordance with WHO guidelines [22], no special restrictions on travel or trade were imposed at any stage of the epidemic outside the very specific areas where cases had been reported. However, some local officials requested people within their jurisdiction not to travel to affected areas. For example, the Health Secretary of Kalangala District, a sparsely inhabited collection of 83 islands in Lake Victoria, asked fishermen from Gulu who were living on the islands not to travel home. “We have no quarrel with people from Gulu,” he said on Day 32. “We are merely appealing to everyone doing fish business with that district to curtail their visits until the situation clears.”

Local officials also played a key role in facilitating the resettlement of stigmatised survivors. As Gulu District Director of Health Services explained after the epidemic was over, “The survivors are not infectious, but the communities took long to accept that. So we had to set up a counselling unit to accompany them into their communities.” As part of the education process, he personally went around shaking hands with survivors in order to demonstrate that they were no longer infectious.

### Regional and international responses: Scientific and financial support, and some travel restrictions

From the start, both the government and WHO were against imposing restrictions on people travelling to or from Uganda. The WHO disease outbreak co-ordinator was quoted on Day 5 as saying: “travel restrictions would be inappropriate because the disease is in a very remote part of Uganda. It spreads by direct contact with bodily fluids, not by sitting next to an infected person on a plane. Cordoning off an area does not work in situations like this.” The message was repeated numerous times throughout the epidemic, but it was not always taken up by other governments, either regionally or further afield.

Neighbouring Kenya responded by sending a “squad of public health officers” on Day 5 to the Busia border post to screen travellers coming in from Uganda. The Busia District Medical Officer said that while they were unable to test for Ebola, their task was to confine those suspected to be suffering from the disease. The policy was not consistently implemented, however, and cases were reported of healthy Ugandans being refused entry to Kenya, and even deported. For example, none of the Ugandans on board a cargo boat that docked on Day 8 at Kisumu port on Lake Victoria were allowed to disembark. Furthermore, on Day 44, the Kenyan government implemented the Epidemic Control Act in order to expel 137 delegates from a meeting in Nairobi of Acholi leaders – half of whom had travelled directly from Gulu – who had come together to discuss building peace in northern Uganda. A Ugandan Ministry of Health official lamented that, “the outbreak should not affect travel out of Uganda, but we cannot tell Kenya what to do.”

Saudi Arabia took a firm position on the epidemic, but not until it was effectively over. Around 300 Ugandan pilgrims had travelled freely to the country while the epidemic was at its height to perform the *Umrah* pilgrimage during Ramadan (in December 2000); but on Day 98 – just five days before the last of the survivors was discharged from hospital – a directive was sent from Riyadh to the Saudi embassy in Kampala, ordering officials to issue no more visas to Ugandans until further notice. In spite of a plea from a senior WHO official in Uganda, the ban stayed in effect throughout the annual Muslim pilgrimage to Mecca, the *Hajj*, in March 2001. As a result, while 600 Ugandan pilgrims had undertaken the pilgrimage in 2000, none managed to do so in 2001.

No travel restrictions were imposed by any European or North American countries. However, a “polite request” was observed by a Ugandan traveller in Oslo airport, asking anyone from Gulu to identify themselves to immigration officials. Belgium also obliged airline passengers from Entebbe to indicate on a form where they had stayed in Uganda, their place of residence in Belgium, their telephone number, and their seat number on the incoming plane.

The reaction of the international scientific and medical community was rapid, with filovirologists from CDC and WHO, and clinicians from Medicins Sans Frontieres in the country by Day 6. A team from the South African National Institute for Virology arrived on Day 41 to catch and test bats, rats, and other rodents for Ebola in an attempt to establish the natural host of the virus.

Likewise, the donor community responded quickly, supplementing by Day 6 the government’s own Ebola budget of 500 million Uganda shillings (US$285,000) with an additional US$400,000. More than 20 international NGOs and government agencies contributed to combating the epidemic [23], providing expertise, cash, protective gear, medicines, vehicles, disinfectant, walkie-talkies, relief food and provisions for survivors. It is difficult accurately to calculate the value of the donated goods from press cuttings, but collectively the reports suggest that it was well in excess of US$3.5 million.

The WHO representative in Uganda commended the efforts of the donor community, saying, “It was as if people thought: ‘there is a disaster somewhere. Let us go and help our brothers’. I have never seen it anywhere else.” However, the donations were not always given purely out of selflessness. As the Irish Junior Foreign Affairs Minister admitted, “this is the sole viable response since there is no specific treatment or vaccine for Ebola. The consequences of an uncontained outbreak would be horrendous.”

### Controversy over the source: Scoring political points

The most politically heated issue of the outbreak concerned where it had come from. Many of the early victims had lived in Aswa County, near Gulu town, where a number of UPDF soldiers, returning from the ongoing war in the DRC, had briefly been settled. As a result, some Gulu residents thought that the soldiers and their newly acquired Congolese wives had brought the disease with them, accusations which were swiftly denied by the army (see also [12]).

However, in an editorial on Day 5, the Monitor suggested that the government ought to use “people and institutions who are credible” – in other words, not members of the UPDF, which itself was being charged with bringing the virus – for the dissemination of information. Otherwise, “they will be suspected of covering up”. The Monitor argued that a partisan response would not carry the same weight in the public’s mind as one issued by, for example, the Ministry of Health. Subsequently, the Minister of Health himself announced that returning UPDF soldiers were not responsible for the outbreak, adding “if any soldier died of Ebola, we would tell you.”

The official position seemed to be vindicated on Day 9, when virologists from the Centres for Disease Control (CDC) announced that they had identified the culpable strain as the Ebola-Sudan variety – as opposed to the other major strain, Ebola-Zaire – which had last been recorded in Nzara, southern Sudan, in 1979. However, according to an opinion column in the Monitor, the announcement was immediately seized upon by “some chaps in government [who] saw this as a gold mine to deliver a political statement”: that LRA rebels based in Sudan – and not the UPDF in the DRC – must therefore have brought the virus. The idea was supported by “unconfirmed” but highly suggestive reports in the New Vision that the rebels had been trading in the brains of baboons and monkeys. On that very same day, however, the rebels themselves released 40 people they had recently abducted in Gulu, apparently because *they* were afraid of catching Ebola. Furthermore, on Day 19, it was reported that they had abandoned one of their transit routes through one of the main Ebola-hit areas.

The actual source of the outbreak will, in all likelihood, never be known. As one of the WHO Ebola specialists explained, central Africa is “endemic for filoviruses… [and] it’s not inconceivable that there has been an Ebola virus in Uganda for some time”. Nonetheless, when the epidemic was finally declared over and the lessons learned were being discussed and analysed, a Monitor editorial took the opportunity to argue that “the best thing is to avoid a situation where diseases are brought by soldiers returning from foreign military adventures, rebels or fleeing refugees. We should have sound politics at home so that we live in peace with our neighbours”.

## Discussion

This paper presents an analysis of responses to a major Ebola outbreak, from the perspectives of the afflicted communities, from Uganda as a whole, as well as from the region and further afield internationally. Previous social science papers have examined experiences from within Ebola-afflicted communities [12,14,15], but events far from the outbreak itself have not been widely reported upon. It is important that such events – including false alarms, the highly politicised debate about the source of the virus, as well as deportations from neighbouring countries are also recognised as part of the broader response.

The over-riding theme of the Uganda Ebola outbreak of 2000/2001 – the largest on record – was fear. This fear led, among many other actions, to people seeking solace in superstitious beliefs (whether traditional, Christian, or Muslim); to health workers quitting their posts; to panic-stricken patients fleeing their hospital beds; and, on at least one occasion, to official authorities battling with each other to avoid picking up a suspected Ebola corpse. These very direct attempts at self-preservation were based either on establishing as much physical distance as possible between the actor and the perceived location of the virus, or on the hope that some higher power might intervene to ensure continued good health. They did not, however, involve any sort of direct social interactions with people or groups who were infected or affected by Ebola. The data presented in this paper suggest that such social interactions were grounded in either altruism or stigma.

### Altruism

Taking the definition of altruism given earlier – an act that “is or appears to be motivated mainly out of a consideration of another’s needs rather than one’s own” (page 30) [19] – a combination of altruistic responses can be seen, ranging from the entirely selfless to those with at least a degree of self-interest. The most selfless actors in this outbreak were clearly those who had, and who *knew* they had, the most to lose – in the form of their lives. These were the health workers, the ambulance drivers, and the burial teams, although even in these cases many would have acted out of a degree of self-interest, in the sense that they possibly stood to lose their jobs should they have defaulted from work. Similarly, the donor community, who flooded the country with technical and financial assistance, did so out an apparently genuine desire to assist, a wish to “help our brothers”; but this too was coupled with a quite explicitly stated concern to prevent the outbreak from spilling over the borders to neighbouring countries and beyond. In this case, the mixing between humanitarianism and self-protection was premised around the principle that ‘your problem is my problem’. It was also easy, politically, to justify expenditure and effort in these altruistic acts, since, as the Irish Minister explained, the consequences of an uncontained outbreak would have been “horrendous”.

### Stigma

The second core social response was stigma, based on a fear of people who were either infected or affected. This stigma manifested itself differently, both qualitatively and quantitatively, according to where it was enacted. At the local level, ‘*they*’ were the victims themselves, the survivors, orphans and other relatives, health workers, and burial teams; while ‘*we*’ could be seen as the uninfected and untouched remainder of the community. The stigma experienced at this level was the most damaging, including cases of people’s huts and belongings being burned; or that experienced by the health workers who, through acting heroically against the fearsome disease, became, in the community’s eyes, the very thing that they were fighting. In many cases, this would have had severe effects on their subsequent “life chances” (as theorised by Goffman [20]).

Nationally, ‘*they*’ were people from Gulu, Masindi and Mbarara, while ‘*we*’ were, once again, the uninfected and untouched remainder of the country; and internationally, ‘*they*’ were all Ugandan nationals, and ‘*we*’ were everyone else in the world. The stigma experienced at these ‘higher’ levels was certainly inconvenient for those who suffered it, including deportation (for example, from Kenya) and other restrictions on movement; but, unlike at the local level, it would probably not have led to any long term impact on the “life chances” of those who had been stigmatised.

### Methodological reflections

The only viable way of studying responses from around the world to an outbreak of Ebola, or indeed of any other virulent infectious disease, is to rely on secondary data sources. It is simply not possible to be present wherever events related to or caused by the outbreak are happening; and the methodological challenges inherent within this must therefore be examined.

This study has used reports from two quite different newspapers as data sources. As stated in the Methods section above, it was not seen as important which newspaper reported on any given story; both were accorded the same ‘weight’ when choosing which stories or quotes to include in the text. This equality is reflected in the relative proportions of quotes from the two newspapers that are included in the text above: 52 (59%) came from the New Vision, and 36 (41%) came from the Monitor (see Additional file 1: Annex 1). These proportions are almost identical to those of the total number of cuttings taken, of which 371 (58%) came from the New Vision and 268 (42%) came from the Monitor. Thus, there is little likelihood of bias caused by over- or under-representation of either newspaper.

A second point concerns the need to rely on other people’s representations of events, which means that there has been no means of verifying what was written unless a given story was reported on by both the New Vision and the Monitor. However, because the articles were produced by many different reporters from the two newspapers, and since they were, for the most part, sober and restrained in tone, it is likely that any bias in perspective would have been diluted. Thus, while this article cannot claim to present an objective depiction of “what really happened” during this outbreak, I am confident that it nonetheless gives a broadly accurate picture of some of the key events.

### Lessons for the future: The importance of openness

With 11 Ebola outbreaks and 348 recorded deaths recorded during the 1990s, and 11 outbreaks causing 725 recorded deaths in the years since 2000 [3], Ebola remains as an ongoing, but, in the grand scheme, relatively minor threat to global health. Nonetheless, it is clear from the Ugandan experience of 2000/2001 that the responses to an outbreak can be very dramatic, and perhaps disproportionate to the actual danger presented. Indeed, in some cases, the responses during this outbreak actively inhibited control and containment measures. An important objective for any future outbreak control strategy must be to prevent excessive fear, which, it is expected, would reduce stigma and other negative outcomes while simultaneously encouraging more altruistic responses.

With regards the provision of public information, it is notable that many of the Ugandan Ministry of Health officials who worked on the 2000/2001 outbreak (for example, Okware et al. [13]; and Lamunu et al. [24]) had years of experience behind them working with AIDS. Thus they were intimately acquainted with the country’s ‘open’ AIDS policy, initiated in the mid-late 1980s, when elected officials from national level all the way down to village level were required by presidential decree to bring AIDS into their discussions at every public meeting [25]. This long-standing official culture of openness clearly affected the way they then dealt with the Ebola outbreak, since they well understood the potential implications if the public sensed that they were hiding or downplaying the full extent of the outbreak. As one of them quoted above explained, “If we hide Ebola, we will kill ourselves. We are very open.”

## Conclusions

Given the extent of the fear and stigma that prevailed throughout the course of the 2000/2001 Ugandan Ebola outbreak, the value of being open in the provision of public information during any future outbreak anywhere in the world – and, critically, of being *seen* to be open – cannot be overstated.

This principle of openness, learned over the long course of the Ugandan AIDS epidemic, helped in the country’s 2000/2001 Ebola outbreak to keep an occasionally panicky populace from excessive over-reaction, and thereby contributed to containing the virus, as well as to reducing stigma and other negative consequences. Countries suffering an Ebola outbreak in the future may well benefit in their attempts to combat the disease by taking on board this important lesson from the Ugandan experience.

## Abbreviations

CDC: Centers for Disease Control; DRC: Democratic Republic of Congo; KCC: Kampala City Council; LRA: Lord’s Resistance Army; UPDF: Uganda People’s Defence Force; WHO: World Health Organization.

## Competing interests

The author declares that he has no competing interests.

## Author’s contributions

JK conceived the idea for the study, collected and analysed all the data, and wrote the paper.

## Author information

The author worked as a behavioural scientist with the Medical Research Council Programme on AIDS in Uganda between 1996 and 2001, and was present in the country for the duration of the 2000/2001 Ebola outbreak. His PhD, conducted at the University of Amsterdam, focused on the influence of evidence and ideology on AIDS policy in Uganda over the course of the epidemic. He is now Deputy Director of the Umeå Centre for Global Health Research, Umeå University, Sweden.

## Supplementary Material

Additional file 1 ** Annex 1.Headlines of newspaper articles quoted in the text.** Click here for file
