# Supplementary material for: “A time of fear”: local, national, and international responses to a large Ebola outbreak in Uganda
Source: Global Health. 2012 Jun 13;8:15. doi: 10.1186/1744-8603-8-15 (PMC3477054; doi:10.1186/1744-8603-8-15)
Supplement: Additional file 1 — Annex 1.Headlines of newspaper articles quoted in the text. [file 1744-8603-8-15-S1.pdf]

Annex 1 – Headlines of newspaper articles quoted in the text

Saturday October 14 – Day 2

- ‘WHO rushes experts to study Gulu fever’ (New Vision)
- ‘Strange disease kills 30 in Gulu’ (Monitor)

Monday October 16 – Day 4

- ‘Ebola death toll goes up’ (New Vision)
- ‘Minister Kiyonga advises on Ebola’ (Monitor)

Tuesday October 17 – Day 5

- ‘Ebola now hits Kitgum’ (New Vision)
- ‘WHO against flight restrictions on Uganda’ (New Vision)
- ‘Kenya screens at border’ (New Vision)
- ‘Open 24 hour Ebola hotline’ (Monitor)
- ‘Residents flee patients, Lira in panic’ (Monitor)

Wednesday October 18 – Day 6

- ‘Ebola experts rush to Lira’ (New Vision)
- ‘US team coming’ (New Vision)
- ‘Ebola now in Soroti, Lira, Atiak’ (Monitor)
- ‘More WHO experts arrive’ (Monitor)

Thursday October 19th – Day 7

- ‘Mambas ferry Ebola fighters’ (New Vision)
- ‘Tororo and Soroti okay’ (New Vision)

Friday October 20th – Day 8

- ‘Ebola now tamed, say doctors’ (New Vision)
- ‘Crew barred’ (New Vision)

Saturday October 21st – Day 9

- ‘Ebola came from Sudan’ (New Vision)
- ‘Ugandan crew on the MV Kabalega was refused to disembark at Kisumu Port in Kenya because of Ebola virus scare’ (New Vision)
- ‘Gulu Ebola from Sudan’ (Monitor)

Sunday October 22nd – Day 10

- ‘Ebola, Embalasasa, and climate of uncertainty’ (Monitor)

<u>Monday October 23rd – Day 11</u>

- ‘Ebola started back in August, US paper says – 54 dead, 149 infected’ (Monitor)

<u>Wednesday October 25th – Day 13</u>

- ‘Ireland joins Ebola war’ (New Vision)
- ‘Ebola cases rise to 165’ (Monitor)

<u>Friday October 27th – Day 15</u>

- ‘Keep politics out of Ebola tragedy’ (Monitor)

<u>Tuesday October 31st – Day 19</u>

- ‘Gulu erupts as local healers chase Ebola’ (Monitor)
- ‘Kony rebels abandon route for fear of Ebola’ (New Vision)

<u>Friday November 3rd – Day 22</u>

- ‘Ebola experts rush to Mbarara’ (New Vision)

<u>Saturday November 4th – Day 23</u>

- ‘15 Ebola suspects in Mbarara hospital’ (Monitor)
- ‘Ebola stops hand shaking’ (New Vision)

<u>Monday November 6nd – Day 25</u>

- ‘Ebola death toll hits 90’ (Monitor)

<u>Tuesday November 7th – Day 26</u>

- ‘Mbarara Ebola cases to 5’ (Monitor)
- ‘Gulu economy fails as Ebola takes toll’ (Monitor)

<u>Friday November 10th – Day 29</u>

- ‘Churches hold Ebola prayers’ (Monitor)
- ‘Ebola: locals threaten team’ (Monitor)

<u>Sunday November 12th – Day 31</u>

- ‘Guns rock Gulu as rebels attack’ (Monitor)

Monday November 13th – Day 32

- ‘Ebola kills 3 in Masindi’ (New Vision)

Tuesday November 14th – Day 33

- ‘Ebola: Kalangala wants Gulu visitors restricted’ (New Vision)
- ‘4th Ebola victim dies in Masindi’ (New Vision)

Wednesday November 15th – Day 34

- ’30 patients flee Kiryandongo over Ebola’ (New Vision)

Thursday November 16th – Day 35

- ‘Ebola claims three more’ (New Vision)
- ‘Belgium screens Ugandans for Ebola’ (Monitor)

Saturday November 18th – Day 37

- ‘Boozer’s vomit causes Ebola scare in taxi’ (New Vision)

Thursday November 23rd – Day 42

- ‘Ebola experts from the South African NIV are to round up and test rats, bats and other rodents in Gulu for the virus (New Vision)

Saturday November 25th – Day 44

- ‘Kacoke Madit blocked’ (New Vision)
- ‘Kenya expels Ugandans over Ebola scare’ (Monitor)

Monday November 27th – Day 46

- ‘Ebola rumours are a positive sign’ (New Vision)

Tuesday November 28th – Day 47

- ‘Ebola kills another 16’ (New Vision)

Wednesday November 29th – Day 48

- ‘Ebola control effort a triumph in teamwork’ (New Vision)

Saturday December 2 – Day 51

- ‘Ebola steals Lacor nurse’ (New Vision)

Tuesday December 5 – Day 54

- ‘Dinsinfecting an Ebola suspect’ (New Vision)
- ‘Masindi family loses 4 to Ebola’ (New Vision)

Wednesday December 6 – Day 55

- ‘Ebola kills Dr Lukiya’ (New Vision)
- ‘MPs want Lukwiya honoured’ (Monitor)

Thursday December 7 – Day 56

- ‘Masindi protests Ebola cemetery’ (New Vision)

Saturday December 9 – Day 58

- ‘Government probes how medics got Ebola’ (Monitor)
- ‘Karimojong ‘cast out’ Ebola’ (Monitor)
- ‘100 patients flee Masindi hospital over Ebola fear’ (New Vision)
- ‘Nsambya in Ebola scare’ (New Vision)
- ‘Ebola scare in hospital as factory workers in gloves bring sick man’ (New Vision)
- ‘Norway’s darkness at noon and Ebola spin’ (Monitor)

Sunday December 10 – Day 59

- ‘Medics vow to shun Ebola victims’ (Monitor)

Monday December 11 – Day 60

- ‘Hospital boss flees threats over Ebola’ (Monitor)
- ‘Ebola scare’ (Monitor)

Tuesday December 12 – Day 61

- ‘Bustling through the streets of Gulu’ (New Vision)
- ‘Muslims cautioned’ (New Vision)

Wednesday December 13 – Day 62

- ‘Bunyoro leaders to meet over Ebola’ (New Vision)

Thursday December 14 – Day 63

- ’30 volunteers for Masindi, Gulu’ (New Vision)

Friday December 15 – Day 64

- ‘Ebola nurses get 816,000 shillings – health boss’ (Monitor)
- ‘Lacor admissions resume’ (New Vision)
- ‘Ebola: Families of health workers to be compensated’ (New Vision)

Saturday December 16 – Day 65

- ‘Pickpockets shun passengers from Gulu, Masindi over Ebola’ (New Vision)

Monday December 18 – Day 67

- ‘Ebola scare’ (Monitor)

Tuesday December 19 – Day 68

- ‘Dead man abandoned at park over Ebola’ (New Vision)

Thursday December 21 – Day 70

- ‘No risk allowance for non-Ebola staff – Opio’ (Monitor)

Tuesday January 16 – Day 96

- ‘Firm aids Ebola war’ (New Vision)

Thursday January 18 – Day 98

- ‘Saudi bans Uganda pilgrims’ (New Vision)

Friday January 19 – Day 99

- ‘WHO pleads for Mecca pilgrims’ (New Vision)

Monday January 22 – Day 102

- ‘Ebola: no more PE for Gulu schools – children are closely monitored and all personal contact is prohibited’ (New Vision)
- ‘Ebola in retreat’ (New Vision)
- ‘Update Ebola radio ads’ (Monitor – letter)

Tuesday January 23 – Day 103

- ‘Ebola survivors tell their tales – whereas the Ebola epidemic is ending medically, social and economic problems are just beginning’ (New Vision)

Wednesday January 24 – Day 104

- ‘Masindi declared Ebola free, last Gulu patient well – outbreak leaves 150 orphans in Gulu’ (Monitor)
- ‘Ebola fever – disease-wary Saudis deny Cranes visas for soccer trip’ (New Vision)

Tuesday January 30 – Day 110

- ‘Hope fades for Mecca pilgrimage’ (New Vision)

Saturday February 24 – Day 135

- ‘End of Ebola: and in memory of the victims’ (Monitor)

Tuesday February 27 – Day 138

- ‘Uganda free of Ebola at last’ (Monitor)
- ‘Uganda declared Ebola free today’ (Monitor)
